# IgA Nephropathy in Native Kidneys: Oxford and Banff Classifications Reveal Distinct Profiles and Predict Outcomes in Pediatric and Adult Patients

**DOI:** 10.3390/life15081231

**Published:** 2025-08-03

**Authors:** Danijel Milivojević, Gorana Nikolić, Björn Tampe, Maja Pecić, Snežana Babac, Dušan Paripović, Gordana Miloševski Lomić, Voin Brković, Marko Baralić, Aleksandar Janković, Petar Đurić, Nataša Stajić, Jovana Putnik, Sanja Radojević Škodrić, Maja Životić

**Affiliations:** 1 Institute of Pathology “Dr. Ðorđe Joannović”, Faculty of Medicine, University of Belgrade, 11000 Belgrade, Serbia; 2 Department of Nephrology and Rheumatology, University Medical Center Göttingen, 37075 Göttingen, Germany; bjoern.tampe@med.uni-goettingen.de; 3 ENT Clinic, Zvezdara University Medical Center, 11000 Belgrade, Serbia; babacsnezana@gmail.com; 4 Faculty of Special Education and Rehabilitation, University of Belgrade, 11000 Belgrade, Serbia; 5 University Children’s Hospital, Faculty of Medicine, University of Belgrade, 11000 Belgrade, Serbia; 6 Clinic of Nephrology, University Clinical Center of Serbia, Faculty of Medicine, University of Belgrade, 11000 Belgrade, Serbia; 7 Clinical Department for Renal Diseases, Zvezdara University Medical Center, Faculty of Medicine, University of Belgrade, 11000 Belgrade, Serbia; 8 The Institute of Mother and Child Health Care of Serbia “Dr. Vukan Čupić”, Faculty of Medicine, University of Belgrade, 11000 Belgrade, Serbia

**Keywords:** IgA nephropathy, Oxford classification, Banff classification, pediatric population, adult population, CKD

## Abstract

IgA nephropathy is the most common primary glomerulonephritis, with pathohistological changes described by the Oxford classification, while the Banff classification is used in transplant pathology. This study included 253 patients with IgA nephropathy in native kidneys, divided into the pediatric (*n* = 105) and adult (*n* = 148) groups. It aimed to examine clinical, and Oxford and Banff morphological parameters in relation to age, correlations of clinical data with pathohistological parameters, and predictors of the disease outcome. Pediatric patients more frequently presented with macroscopic hematuria, while adults showed higher urea and creatinine levels, and lower eGFR. Examining Oxford classification parameters, chronic glomerular and tubulointerstitial lesions were more common in adults. Banff parameters revealed more frequent chronically active glomerular, inflammatory, chronic tubulointerstitial, and vascular lesions in adults. All inflammatory, chronic tubulointerstitial, and vascular parameters correlated with serum urea levels, eGFR and CKD stage in adults, while less frequent in pediatric patients. Tubulointerstitial Oxford and Banff parameters were strong predictors of CKD and proteinuria progression in children, while such predictors were fewer in adults; segmental glomerulosclerosis predicted hematuria progression in adults. Banff parameters (cg, t, ti, i, i-IFTA, ptc, cv), not in Oxford classification, significantly predict outcomes and are recommended for incorporation into IgA nephropathy reports.

## 1. Introduction

Glomerulonephritides represent a variable group of rare, immune-mediated diseases primarily characterized by glomerular injury [1]. IgA nephropathy is the most common primary glomerulonephritis diagnosed through kidney biopsy [2]. The disease most frequently occurs and is diagnosed in the second and third decades of life [3]. Its clinical presentation is highly heterogeneous: most commonly asymptomatic with findings of microhematuria and subnephrotic proteinuria, as well as episodes of macroscopic hematuria associated with respiratory or gastrointestinal infection. However, it may also manifest as nephritic syndrome, nephrotic syndrome, rapidly progressive glomerulonephritis, or acute kidney injury [4]. Approximately 20% of pediatric patients progress to end-stage chronic kidney disease (CKD) within the first 20 years of illness, while about 30% of adult patients reach this stage 10 to 20 years after diagnosis [5,6].

The gold standard for diagnosing IgA nephropathy is the histopathological evaluation of kidney biopsy specimens [7]. While findings under light microscopy are highly heterogeneous, immunofluorescence typically reveals dominant or co-dominant mesangial IgA deposits, most commonly accompanied by C3 deposition, *λ* light chains and, to a variable extent, IgM and/or IgG [8]. For the light microscopy analysis of biopsy samples, the IgA Nephropathy Classification Working Group recommends using the Oxford classification, which considers the presence of mesangial (M) and endocapillary hypercellularity (E), segmental glomerulosclerosis (S), chronic tubulointerstitial lesions (T), as well as cellular and fibrocellular crescents (C) [9]. However, IgA nephropathy cannot be distinguished histopathologically from nephritis occurring in the context of IgA vasculitis, as both conditions exhibit similar pathological features [10]. Therefore, the distinction is based on clinical presentation. If additional clinical signs and symptoms of vasculitis are present, such as palpable purpura, arthritis/arthralgia, and abdominal pain, IgA vasculitis is the more likely diagnosis. In cases where the disease is limited to the kidneys, the appropriate term is IgA nephropathy [11]. According to literature data, systemic involvement is more frequently observed in the pediatric population [12].

In contrast to the Oxford classification, which primarily focuses on glomerular changes and includes only a single parameter for tubulointerstitial involvement (T), the Banff classification, which is routinely applied in the evaluation of kidney transplant biopsies, providing a comprehensive assessment of glomerular, tubulointerstitial, and vascular changes, offers a more precise and structured assessment [13]. It places particular emphasis on inflammatory lesions across all renal compartments, including not only the glomeruli but also the tubules, interstitium, and blood vessels, whereas many of these inflammatory injury patterns are not routinely analyzed in native kidney biopsies. Although existing literature reports associations between tubulointerstitial changes (such as total inflammation and interstitial fibrosis) and levels of creatinine and proteinuria, similar data on the application and relevance of Banff classification parameters in IgA nephropathy remain limited [14].

The cornerstone of IgA nephropathy treatment is supportive therapy. This includes limiting daily salt intake, smoking cessation, weight control, and physical activity. Pharmacological treatment involves ACE inhibitors or angiotensin receptor blockers to manage blood pressure and reduce proteinuria [15]. In adult patients whose proteinuria remains above 1 g/24 h after three months of optimal supportive therapy and if their estimated glomerular filtration rate (eGFR) is ≥30 mL/min/1.73 m^2^, systemic corticosteroid therapy should be introduced [16]. In contrast, for pediatric patients, glucocorticoid immunosuppressive therapy is initiated alongside supportive treatment immediately after diagnosis if proteinuria exceeds 1 g/24 h, the urine protein-to-creatinine ratio is >1 g/g, and/or mesangial hypercellularity is observed on histopathological evaluation [16]. In patients with a rapidly progressive clinical course, corticosteroid therapy is combined with cyclophosphamide [16,17]. Among the clinical parameters most strongly associated with the risk of progression of CKD are eGFR at the time of biopsy, as well as the levels of proteinuria and arterial blood pressure [18,19]. Literature also highlights the significance of hematuria as an independent factor in the progression of CKD. Moreover, the Oxford classification scores M1, T1, and T2 are independent prognostic factors for the progression of CKD in patients with IgA nephropathy and should be combined with appropriate clinical parameters when assessing disease prognosis, as well as when deciding on the possible early use of corticosteroid therapy [20,21,22,23,24].

Given the different approaches to treating pediatric and adult patients with IgA nephropathy, the aim of this study was to examine, in relation to patient age, both clinical and histopathological parameters of the Oxford and Banff classifications, as well as to correlate each clinical parameter with each pathohistological parameter assessed by both Oxford and Banff classifications. Moreover, in order to define either clinical or histopathological predictors of the disease outcomes, defined as progression to advanced CKD stages, the progression of proteinura and hematuria, the patients were followed up, and the data was collected at the last control medical examination.

## 2. Materials and Methods

### 2.1. Patients

This retrospective study included 253 patients diagnosed with IgA nephropathy in native kidneys during the period from 2005 to 2024 at the Institute of Pathology “Dr. Đorđe Joannović” of the Faculty of Medicine, University of Belgrade. The initial diagnosis was made on fresh-frozen kidney sections based on the dominant or co-dominant mesangial IgA deposits, accompanied by C3 and *λ* light chains deposition. The patients were treated at the Clinic of Nephrology of the University Clinical Center of Serbia, the Emergency Center of the University Clinical Center of Serbia, Zvezdara University Medical Center, the University Children’s Hospital and the Institute of Mother and Child Health Care of Serbia “Dr. Vukan Čupić”. All patients were screened for viral infection markers, (hepatitis B, hepatitis C, and HIV), as well as for antistreptolysin O (ASO) titers; those test results were negative. The patients without available medical records were not included. The participants were divided into two groups according to age: the pediatric group (*n* = 105) and the adult group (*n* = 148). Patients younger than 18 years were classified as pediatric population, while those older were considered adults.

### 2.2. Clinical and Laboratory Data

Laboratory and clinical data, recorded at the time of biopsy and at the time of last control medical examination, were collected through a review of the patients’ medical records. In the pediatric population, proteinuria values of 4–40 mg/m^2^/h were classified as subnephrotic, and values >40 mg/m^2^/h as nephrotic [25]. In the adult population, values of 0.15–3.49 g/24 h were classified as subnephrotic, and ≥3.5 g/24 h as nephrotic [16]. The physiological range of serum albumin is 35–52 g/L, for total proteins 62–82 g/L, and for serum urea 2.1–7.1 mmol/L [26,27]. The stage of CKD was determined based on the eGFR [16].

### 2.3. Treatment Protocols

The primary approach to treatment included the administration of ACE inhibitors, with or without angiotensin receptor antagonists, aiming to reduce proteinuria and control blood pressure. In adult patients, SGLT2 inhibitors were also commonly prescribed as part of supportive therapy; dapagliflozin was used at a daily dose of 10 mg [28,29]. Pediatric patients received glucocorticoid therapy immediately upon diagnosis: when proteinuria exceeded 1 g/24 h, the urine protein-to-creatinine ratio was greater than 1 g/g, or mesangial hypercellularity was identified via biopsy. Conversely, systemic corticosteroids were initiated in adults if their proteinuria remained above 1 g/24 h despite three months of optimized conservative management, but whose eGFR was ≥30 mL/min/1.73 m^2^.

Corticosteroid treatment protocols have evolved over time. Administration according to the Pozzi protocol involved intravenous bolus injections of 1 g methylprednisolone for 3 consecutive days during months 1, 3, and 5, followed by oral prednisone at a dose of 0.5 mg/kg on alternate days for a total of 6 months [30]. The Manno protocol involved a 6-month oral prednisone treatment, beginning with a dose of 0.8–1 mg/kg/day for the initial 2 months, then tapering the dose by 0.2 mg/kg/day each month over the following 4 months [31]. In line with the protocol described by Lv et al., prednisone was given at 0.8–1.0 mg/kg/day for 8 weeks, followed by a gradual dose decrease of 5–10 mg every two weeks, resulting in a total treatment period of about 8 months [32].

### 2.4. Pathohistological Analysis

All biopsy specimens were independently reviewed by three pathologists. An initial assessment of the biopsy sample quality was performed to determine the eligibility for further analyses. In accordance with the Oxford classification, which requires a minimum of 8 viable glomeruli for accurate MEST-C scoring, biopsy specimens with fewer than 8 viable glomeruli were excluded, resulting in a final cohort of 194 samples for further pathohistological analysis. The light microscopy analysis of kidney biopsy specimens was performed using hematoxylin–eosin (HE), periodic acid–Schiff (PAS), and Masson’s trichrome and Jones methenamine silver (JMS) stains. The following parameters of the Oxford classification were assessed: mesangial hypercellularity (M), endocapillary hypercellularity (E), segmental glomerulosclerosis (S), tubular atrophy/interstitial fibrosis (T), and cellular/fibrocellular crescents (C) [9]. Additionally, using Banff classification scheme, the following features were evaluated: in the glomeruli–glomerulitis (g), glomerular basement membrane double contours (cg) and mesangial matrix expansion (mm); in the tubulointerstitium—tubulitis (t), total inflammation (ti), interstitial inflammation (i), inflammation in the area of interstitial fibrosis and tubular atrophy (i-IFTA), tubular atrophy (ct), and interstitial fibrosis (ci); in the blood vessels—peritubular capillaritis (ptc), vascular fibrous intimal thickening (cv), arteriolar hyalinosis (ah), and hyaline arteriolar thickening (aah) [13].

### 2.5. Statistical Analysis

Statistical analysis was performed using the IBM SPSS Statistics 25 software (SPSS Inc., Chicago, IL, USA). To assess data normality, computational methods were applied: the coefficient of variation, skewness, and kurtosis values. The statistical significance of differences in the frequencies of nominal and ordinal variables between the pediatric and adult populations was assessed using the *χ*^2^ test for rxk tables and Fisher’s exact test. Differences in the values of the numerical variables between the study groups were evaluated using the Student’s *t*-test for two independent samples for data following a normal distribution, or the Mann–Whitney U test for data not following a normal distribution. In order to evaluate the strength and direction of correlations between clinical data and Oxford and Banff classification parameters, Spearman’s rank correlation coefficient was used. Hypotheses were tested at a significance level of 0.050.

In order to define the predictors of the disease outcome, univariate analysis was performed applying Kaplan–Meier estimator. Three outcomes were initially defined. The first outcome referred to the progression of CKD, defined as a transition from a lower to a higher CKD stage from the time of kidney biopsy to the last control medical examination, with the CKD stage determined based on eGFR. The second outcome was defined as proteinuria progression, assessed by comparing its value at the last follow-up with the level at the time of biopsy. The progression of proteinuria was defined as an increase in its value between these two defined time points. The third one was defined as hematuria progression, based on a comparison of its presence at the time of kidney biopsy and at the last nephrology follow-up. Progression was defined as the presence of hematuria at the final follow-up in a patient who had no hematuria at the time of biopsy, or a change from microscopic hematuria at the time of biopsy to macroscopic hematuria at the last control medical examination.

All data collected at the time of kidney biopsy, including clinical and laboratory findings, as well as morphological parameters based on the Oxford and Banff classifications, were considered potential predictive factors. For the purpose of univariate analysis, continuous numerical variables were categorized using established physiological cut-off values for serum total protein, serum albumin, serum urea, and eGFR. Comparative analysis between patients with and without adverse outcomes was conducted using the two-sided log-rank test. Variables demonstrating statistical significance (*p* < 0.050) in univariate analysis were considered potential predictors for the development of kidney function decline.

## 3. Results

### 3.1. Clinical Parameters in Pediatric and Adult IgA Nephropathy

A detailed presentation of clinical parameters among pediatric (*n* = 105) and adult (*n* = 148) subjects is provided in Table 1. Of the total number of patients, 66% (*n* = 168) were male, while the remaining 34% (*n* = 85) were female. Among those presenting with symptoms and signs of IgA vasculitis, a large proportion were pediatric patients, comprising 71% (*n* = 52) of the cohort. This distribution was statistically significant (*p* < 0.001) and reflects the higher frequency of systemic involvement in the pediatric population. While adults typically presented with either no hematuria or microscopic hematuria, macroscopic hematuria was the predominant finding in children, occurring in 72% (*n* = 48) of pediatric cases (*p* < 0.001). In both adults and children, proteinuria was a common finding, most often in the subnephrotic range, and did not differ significantly between the groups (*p* = 0.383).

Although serum total protein and albumin levels tended to be closer to the lower limit of the reference range, no statistically significant differences were observed between the two groups. In contrast, nitrogenous waste products (serum urea and creatinine) were significantly elevated in the adult population compared to the pediatric group (*p* = 0.017 for urea; *p* < 0.001 for creatinine). The eGFR was significantly higher in children (117 ± 44.3 mL/min) than in adults (71 ± 37.2 mL/min) (*p* < 0.001). Accordingly, a significantly greater proportion of adult patients were classified into higher stages of CKD (*p* = 0.012).

The most frequently observed comorbidities in the study population were hypertension and diabetes mellitus. Hypertension was present in 89 individuals, of whom 93% (*n* = 83) were adults and only 7% (*n* = 6) were pediatric patients (*p* < 0.001). In contrast, diabetes mellitus was documented in just eight patients, all of whom belonged to the adult group (*p* = 0.022).

Adults with biopsy confirmed IgA nephropathy were more frequently treated with SGLT2 inhibitors and RAAS inhibitors (*p* < 0.001), whereas corticosteroids were administered in both pediatric and adult patients (Table 1).

### 3.2. Pathohistological Parameters

**Figure 1:**
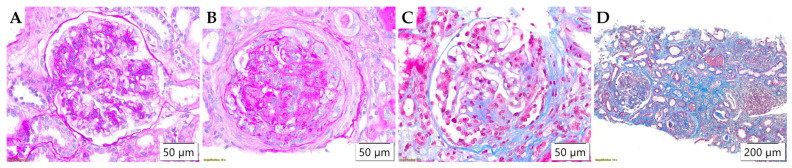
Morphological parameters assessed by Oxford classification. (**A**) Mesangial hypercellularity (M), PAS ×400; (**B**) Endocapillary hypercellularity (E) and cellular crescent (C), PAS ×400; (**C**) Segmental glomerulosclerosis (S), Masson’s trichrome ×400; (**D**) Interstitial fibrosis and tubular atrophy (T), Masson’s trichrome ×100.

The assessment of the Oxford classification parameters revealed that in almost all analyzed samples, mesangial hypercellularity was present in >50% of glomeruli (M1) (Table 2), while endocapillary hypercellularity (E) and crescent formation (C) were frequently observed in adults, but without statistically significant difference. However, chronic lesions, both in glomeruli and tubulointerstitium, were frequently detected in adult onset IgA nephropathy, and accordingly, segmental glomerulosclerosis (S) (*p* = 0.017) and tubular atrophy/interstitial fibrosis (T) (*p* < 0.001) reached a statistically significant difference. Aforementioned morphological parameters assessed by Oxford classification are illustrated in Figure 1.

**Figure 2:**
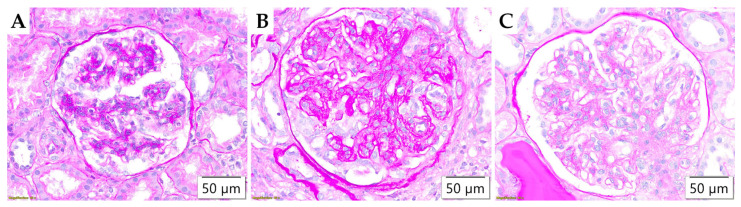
Morphological parameters in glomeruli assessed by Banff classification. (**A**) Glomerulitis (g), PAS ×400; (**B**) Glomerular basement membrane double contours (cg), PAS ×400; and (**C**) Mesangial matrix expansion (mm), PAS ×400.

Glomerular lesions evaluated according to Banff classification are illustrated in Figure 2. The prevalence of glomerulitis (g) and mesangial matrix expansion (mm) were not statistically different between the study populations, while the duplication of the glomerular basement membrane (cg) demonstrated a statistically significant difference in distribution (*p* = 0.047) and was commonly detected in adults (Table 3).

**Figure 3:**
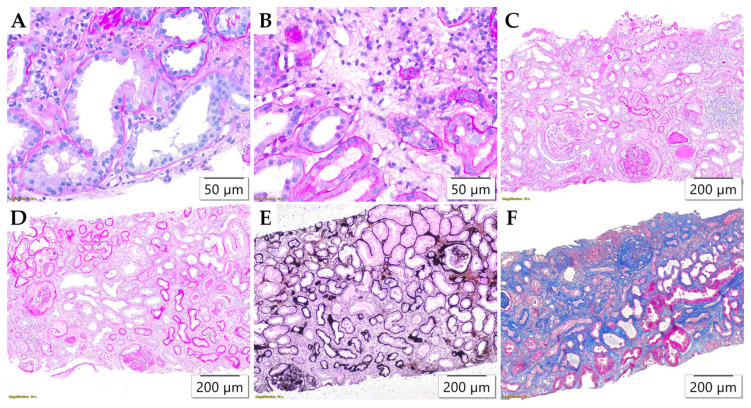
Morphological parameters in tubulointerstitium assessed by Banff classification. (**A**) Tubulitis (t), PAS ×400; (**B**) Interstitial inflammation (i), PAS ×400; (**C**) Inflammation in the area of interstitial fibrosis and tubular atrophy (i-IFTA), PAS ×100; (**D**) Interstitial fibrosis (ci) and tubular atrophy (ct), PAS ×100; (**E**) Interstitial fibrosis (ci) and tubular atrophy (ct), JMS ×100; (**F**) Interstitial fibrosis (ci) and tubular atrophy (ct), Masson’s trichrome ×100.

In contrast to glomerular changes, all the examined Banff parameters related to tubulointerstitial alterations revealed a statistically significant difference in distribution between the study populations (Table 4). Tubulitis (Figure 3A), as an active lesion in the tubules, was almost exclusively recorded in adult patients (*p* < 0.001), more often as mild, t1 (*n* = 18) and moderate, t2 (*n* = 8) than severe, t3 (*n* = 2). Parameters of interstitial inflammation (total inflammation (ti), interstitial inflammation (i) and the inflammation in the area of interstitial fibrosis and tubular atrophy (i-IFTA)), shown in Figure 3B,C, indicated a greater extent of cortical involvement in adults compared to pediatric subjects (*p* < 0.001 for all parameters). In pediatric patients, such changes were rarely observed, and if present, mostly mild or occasionally moderate (ti1, ti2, i1, i-IFTA1, and i-IFTA2), whereas severe interstitial inflammatory lesions (ti3, i3, and i-IFTA3) were observed exclusively among adult subjects.

The distribution of chronic tubulointerstitial lesions, the morphology of which is presented in Figure 3D–F, was also uneven between the two groups (Table 4). Tubular atrophy involving >50% of cortical tubules (ct3) was found exclusively in adult subjects (*p* < 0.001). In pediatric biopsies, interstitial fibrosis most often involved ≤5% of cortical parenchyma (ci0), and only in a small number (*n* = 16) was mild (ci1) or moderate (ci2). Conversely, among adult subjects, interstitial fibrosis of a severe degree (ci3) was also recorded (*p* < 0.001).

**Figure 4:**
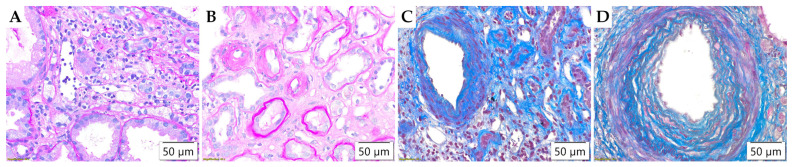
Morphological parameters in blood vessels assessed by Banff classification. (**A**) Peritubular capillaritis (ptc), PAS ×400; (**B**) Arterioral hyalinosis (ah, aah), PAS ×400; (**C**) Vascular fibrous intimal thickening (cv2), Masson’s trichrome ×400; (**D**) Vascular fibrous intimal thickening (cv3), Masson’s trichrome ×400.

A detailed analysis of blood vessels in biopsy samples revealed no evidence of intimal arteritis (v), assessed by Banff. The Banff parameter ptc (shown in Figure 4A), which considers the number of leukocytes in the most affected peritubular capillary, showed a statistically significant difference between study groups (*p* = 0.022), and it was usually detected in adult onset IgA nephropathy in native kidneys (Table 5). The fibrous intimal thickening of blood vessels (Figure 4C,D) was observed in only three pediatric subjects, primarily of mild degree (cv1), whereas moderate (cv2) and severe (cv3) changes were significantly more common in adult subjects (*p* = 0.004). Two Banff parameters related to arteriolar hyalinosis (ah, aah) (Figure 4B) showed statistically significant differences in distribution (*p* < 0.001), as they were mostly observed in adult patients (Table 5).

### 3.3. Correlation of Clinical Data Collected at the Time of Biopsy with Pathohistological Parameters

Correlations between relevant clinical and laboratory parameters and histopathological features were assessed separately in pediatric and adult patients with IgA nephropathy in native kidneys, based on both the Oxford MEST-C and Banff classification systems (Table 6 and Table 7).

According to the Oxford MEST-C scores, mesangial hypercellularity (M) showed no correlation with clinical parameters. Endocapillary hypercellularity (E) was associated with proteinuria levels, CKD stage, and reduced eGFR, but only in the adult population. Segmental glomerulosclerosis (S) correlated with proteinuria in the pediatric group, whereas in adults, it was associated with reduced eGFR. Tubular atrophy/interstitial fibrosis (T) showed significant correlations with CKD stage and eGFR in both pediatric and adult patients. In addition, in the adult group, this lesion also correlated with lower serum albumin and higher serum urea levels. Crescentic lesions (C) were significantly associated with serum albumin levels in both age groups, with a stronger correlation observed in the pediatric population. In adults, the C score also correlated with proteinuria, CKD stage, and eGFR, while in children it was associated with total serum protein and urea levels.

Within the Banff classification, glomerulitis (g) was the only glomerular lesion that significantly correlated with proteinuria, serum albumin, CKD stage, and eGFR, and this correlation was observed exclusively in the adult population.

Among tubulointerstitial Banff parameters in adults, interstitial inflammation in the non-scarred cortex (i), tubulitis (t), interstitial inflammation in the area of interstitial fibrosis and tubular atrophy (i-IFTA), and tubular atrophy (ct) all showed significant correlations with serum urea, reduced eGFR, and CKD stage. In contrast, in the pediatric group, tubulitis (t) showed no correlation with clinical parameters, i-IFTA, and total inflammation (ti) were not associated with urea levels, tubular atrophy (ct) showed no correlation with eGFR or CKD stage, and peritubular capillaritis (ptc) was not correlated with serum urea levels.

As for vascular lesions, fibrous intimal thickening (cv), arteriolar hyalinosis (ah), and arteriolar hyaline thickening (aah) significantly correlated with serum urea, reduced eGFR, and CKD stage in the adult group. Moreover, both ah and aah scores were associated with proteinuria in adults, while in the pediatric population they were linked to hematuria.

### 3.4. Clinical Predictors of the Outcome in Pediatric and Adult Onset IgA Nephropathy in Native Kidneys

Out of 253 patients, 157 were followed for a period of 46 ± 37.0 months (median: 38 months), and their clinical data were collected at the time of their last follow-up examination. To evaluate potential predictors of CKD stage, as well as proteinuria and/or hematuria progression, clinical parameters were analyzed using univariate Kaplan–Meier analysis, separately for pediatric and adult patients with IgA nephropathy in native kidneys.

During the follow-up period, only 10% of pediatric patients showed progression to the advanced CKD stages, whereas 40% of adult patients experienced CKD progression (Table 8). Conversely, both pediatric and adult cohorts demonstrated a reduction in proteinuria and hematuria over the course of the disease (Table 8).

**Figure 5:**
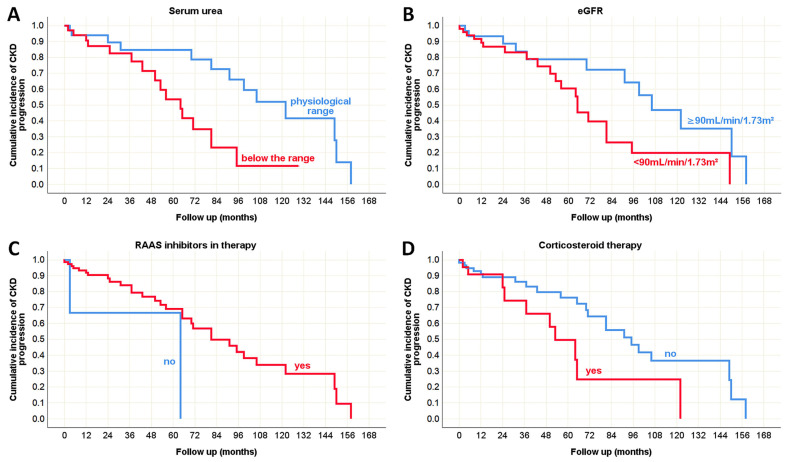
Kaplan–Meier survival curve illustrating clinical predictors of CKD progression in adult onset IgA nephropathy in native kidneys. (**A**) The influence of serum urea values on the CKD progression; (**B**) The influence of eGFR on the CKD progression; (**C**) The influence of RAAS inhibitors in therapy on the CKD progression; and (**D**) The influence of corticosteroid therapy on the CKD progression.

No clinical predictors of disease progression were identified in the pediatric population. However, several predictors were observed in adults with IgA nephropathy (Table 9). Kaplan–Meier survival curves (Figure 5A,B) highlight the positive influence of normal serum urea and eGFR values, recorded at the time of biopsy, on the preservation of normal renal excretory function during follow-up.

Furthermore, the use of RAAS inhibitors was associated with a significant attenuation of CKD progression over time (Figure 5C). In contrast, adult patients who received corticosteroid therapy exhibited faster progression (Figure 5D), although they were initially diagnosed with severe renal impairment, including chronic tubulointerstitial lesions and glomerular crescent formation.

**Figure 6:**
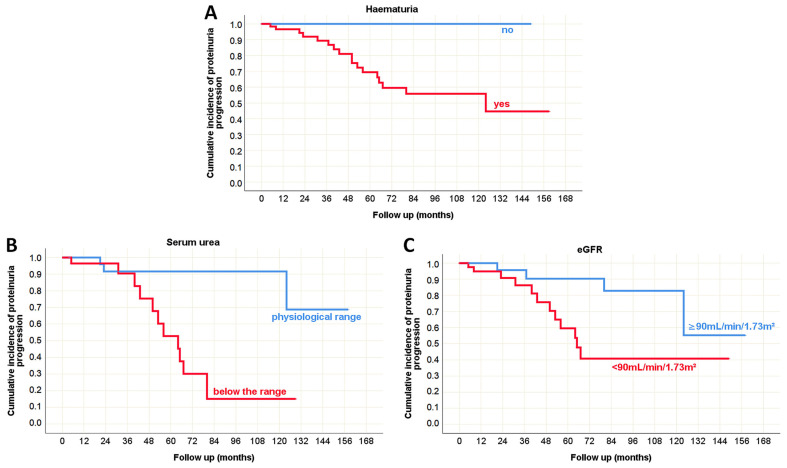
Kaplan–Meier survival curve illustrating clinical predictors of proteinuria progression in adult onset IgA nephropathy in native kidneys. (**A**) The influence of hematuria on the proteinuria progression; (**B**) The influence of serum urea values on the proteinuria progression; (**C**) The influence of eGFR on the proteinuria progression.

Although the majority of patients showed a regression in proteinuria values over the course of the disease (Table 8), a subset of patients exhibited progression. In this minority group, hematuria, serum urea, and eGFR values recorded at the time of biopsy were shown to be potential predictors of proteinuria progression, as illustrated by the Kaplan–Meier survival curve (Figure 6).

### 3.5. Pathohistological Predictors of the Outcome in Pediatric and Adult Onset IgA Nephropathy in Native Kidneys

**Figure 7:**
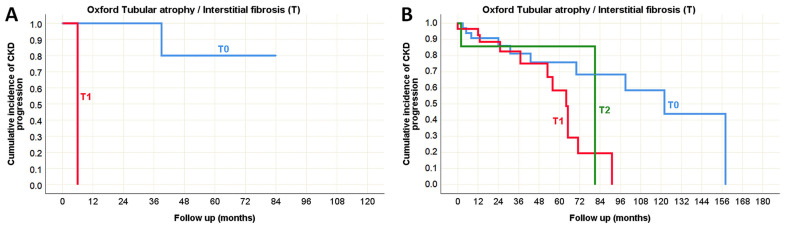
Kaplan–Meier survival curve illustrating Oxford tubular atrophy/interstitial fibrosis (T) as a predictor of CKD progression in (**A**) pediatric and (**B**) adult onset IgA nephropathy in native kidneys.

**Figure 8:**
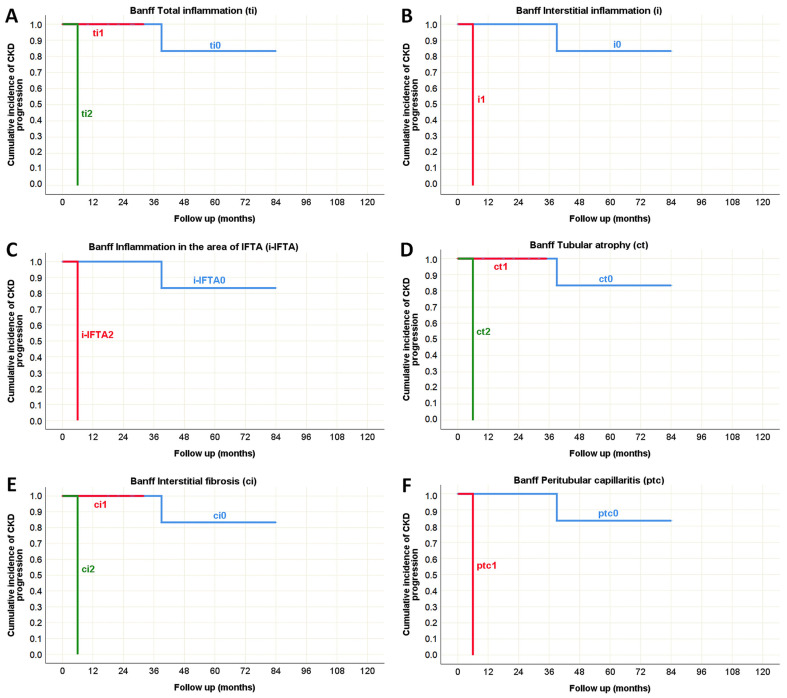
Kaplan–Meier survival curve illustrating Banff classification parameters as predictors of CKD progression in pediatric onset IgA nephropathy in native kidneys. (**A**) The influence of total inflammation (ti) on the CKD progression; (**B**) The influence of interstitial inflammation (i) on the CKD progression; (**C**) The influence of inflammation in the area of IFTA (i-IFTA) on the CKD progression; (**D**) The influence of tubular atrophy (ct) on the CKD progression; (**E**) The influence of interstitial fibrosis (ci) on the CKD progression; (**F**) The influence of peritubular capillaritis (ptc) on the CKD progression.

The univariate analysis of morphological parameters from the Oxford classification revealed that only tubular atrophy/interstitial fibrosis (T) could serve as a predictor of CKD progression in both pediatric and adult-onset IgA nephropathy (Table 10), as illustrated by the Kaplan–Meier survival curve in Figure 7. Additionally, several morphological parameters from the Banff classification were significantly associated with impaired renal function over the follow-up period, indicating that potential predictors of CKD progression were predominantly related to the tubulointerstitial compartment (including ti, i, i-IFTA, ct, ci, and ptc), particularly in the pediatric population (Table 10). It was observed that the absence of these morphological changes in pediatric IgA nephropathy was associated with preserved renal function for at least three years following biopsy, whereas their presence correlated with a decline in eGFR within six months after the biopsy, as shown in the Kaplan–Meier survival curve in Figure 8.

**Figure 9:**
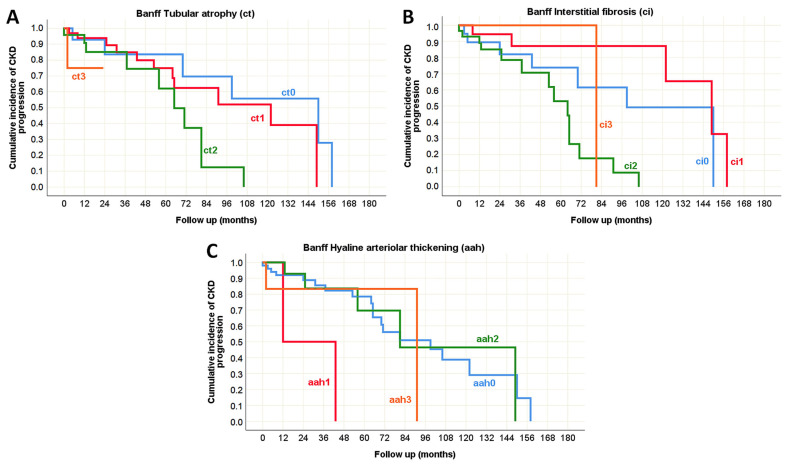
Kaplan–Meier survival curve illustrating Banff classification parameters as predictors of CKD progression in adult onset IgA nephropathy in native kidneys. (**A**) The influence of tubular atrophy (ct) on the CKD progression; (**B**) The influence of interstitial fibrosis (ci) on the CKD progression; (**C**) The influence of hyaline arteriolar thickening (aah) on the CKD progression.

While vascular alterations were not identified as predictors of CKD progression in pediatric IgA nephropathy (Table 10), in the adult group, hyaline arteriolar thickening (aah) emerged as a significant predictor, alongside tubular atrophy (ct) and interstitial fibrosis (ci), as presented in Figure 9.

**Figure 10:**
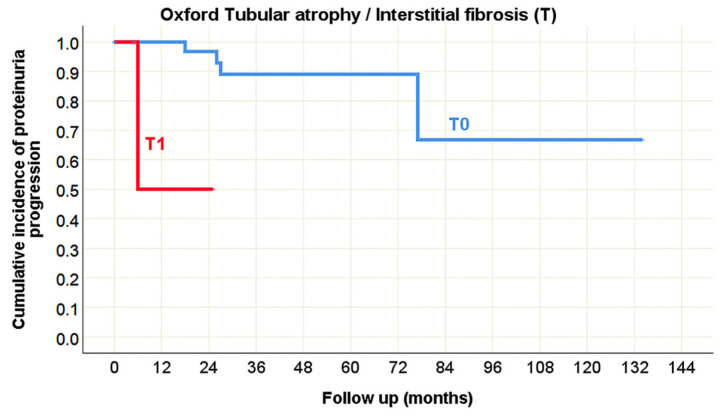
Kaplan–Meier survival curve illustrating Oxford tubular atrophy/interstitial fibrosis (T) as a predictor of proteinuria progression in pediatric onset IgA nephropathy in native kidneys.

**Figure 11:**
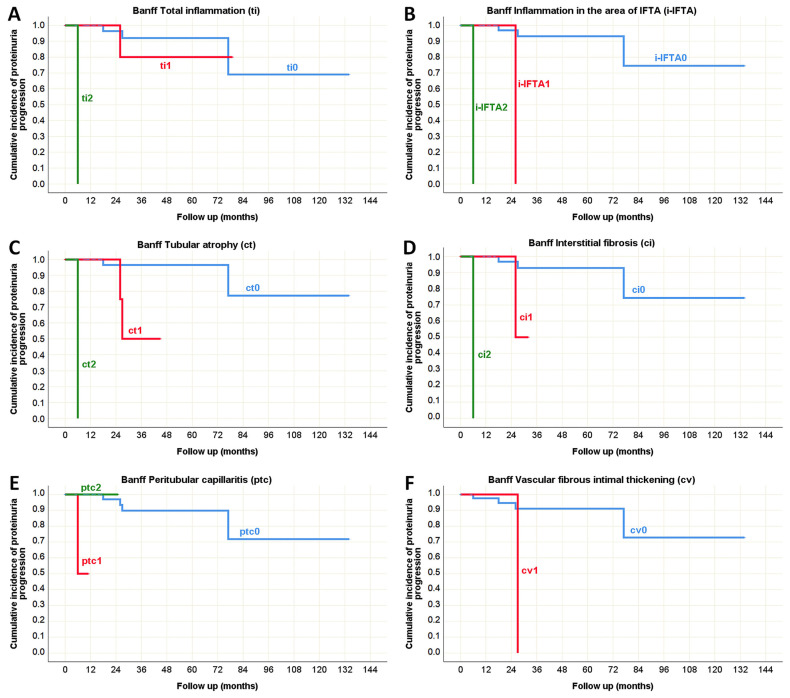
Kaplan–Meier survival curve illustrating Banff classification parameters as predictors of proteinuria progression in pediatric onset IgA nephropathy in native kidneys. (**A**) The influence of total inflammation (ti) on proteinuria progression; (**B**) The influence of inflammation in the area of IFTA (i-IFTA) on the proteinuria progression; (**C**) The influence of tubular atrophy (ct) on the proteinuria progression; (**D**) The influence of interstitial fibrosis (ci) on the proteinuria progression; (**E**) The influence of peritubular capillaritis (ptc) on the proteinuria progression; (**F**) The influence of vascular fibrous intimal thickening (cv) on the proteinuria progression.

**Figure 12:**
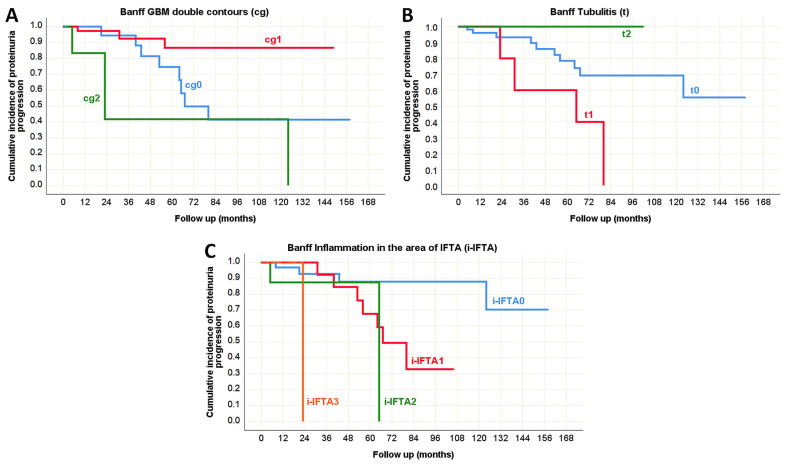
Kaplan–Meier survival curve illustrating Banff classification parameters as predictors of proteinuria progression in adult onset IgA nephropathy in native kidneys. (**A**) The influence of GBM double contours (cg) on the proteinuria progression; (**B**) The influence of tubulitis (t) on the proteinuria progression; (**C**) The influence of inflammation in the area of IFTA (i-IFTA) on the proteinuria progression.

Although proteinuria progression was not frequently observed, in the pediatric population its predictors largely overlapped with those of CKD progression (Table 10), as depicted in Figure 10 (Oxford classification) and Figure 11 (Banff classification). The only exception was interstitial inflammation (i), which was not identified as a predictor of proteinuria progression. Interestingly, vascular fibrous intimal thickening (cv) emerged as a new predictor in this context. In adults, the presence of glomerular basement membrane double contours (cg), tubulitis (t), and inflammation in the area of interstitial fibrosis and tubular atrophy (i-IFTA) were identified as Banff morphological parameters significantly associated with proteinuria progression during the disease course (Table 10, Figure 12).

**Figure 13:**
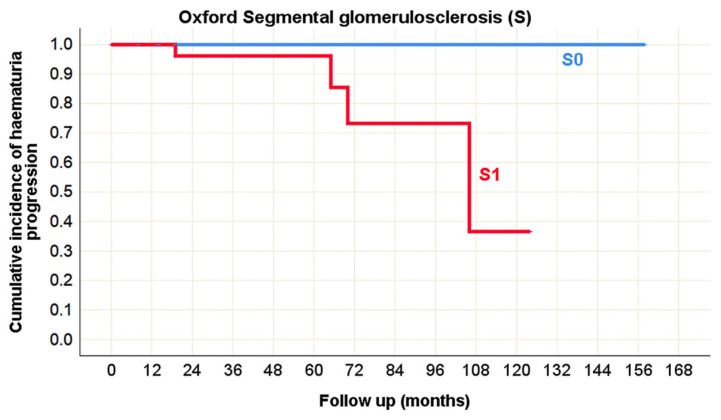
Kaplan–Meier survival curve illustrating Oxford segmental glomerulosclerosis (S) as a predictor of hematuria progression in adult onset IgA nephropathy in native kidneys.

**Figure 14:**
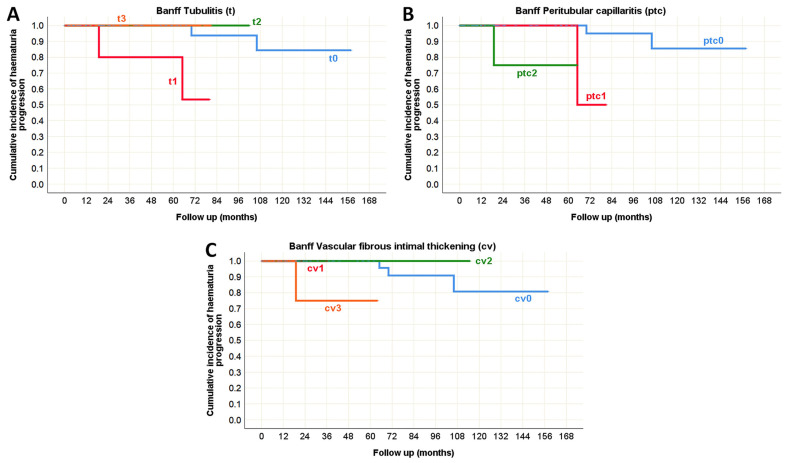
Kaplan–Meier survival curve illustrating Banff classification parameters as predictors of hematuria progression in adult onset IgA nephropathy in native kidneys. (**A**) The influence of tubulitis (t) on the hematuria progression; (**B**) The influence of peritubular capillaritis (ptc) on the hematuria progression; (**C**) The influence of vascular fibrous intimal thickening (cv) on the hematuria progression.

There were no predictors of hematuria progression identified in the pediatric population. However, in adults, several histopathological lesions were associated with hematuria progression during the course of IgA nephropathy in native kidneys. According to the Oxford classification, segmental glomerulosclerosis (S) was recognized as a significant predictor. In the Banff classification, tubulitis (t), peritubular capillaritis (ptc), and vascular fibrous intimal thickening (cv) were identified as morphological parameters significantly correlated with hematuria progression (Table 10, Figure 13 and Figure 14).

## 4. Discussion

This retrospective study analyzed and compared the distribution of a wide range of clinical and pathohistological parameters between pediatric and adult patients diagnosed with IgA nephropathy. Furthermore, the study examined the correlations between pathohistological findings and clinical features, and identified potential predictors of CKD progression, as well as of proteinuria and hematuria progression. In light of the clinical parameters evaluated, existing literature underscores the prognostic relevance of proteinuria levels, arterial blood pressure, and the eGFR at the time of biopsy as key determinants of renal function, particularly in relation to the progression of CKD [18,19]. Beyond the control of arterial hypertension, the reduction in proteinuria represents a very important therapeutic objective. Our study did not demonstrate proteinuria at the time of biopsy as a significant predictive factor for the progression of CKD. However, it did identify eGFR as a significant predictor of both the risk of CKD progression and the worsening of proteinuria levels in adult onset IgA nephropathy.

The literature also highlights the significance of hematuria as an independent factor in the progression of CKD [33]. Hematuria may be microscopic or macroscopic, with macroscopic hematuria observed in approximately 88% of pediatric and 20% of adult patients [34]. The results of our study also demonstrated a higher frequency of hematuria among pediatric subjects. Furthermore, the results indicated that the presence of hematuria in adult patients may serve as a predictor of worsening proteinuria over the course of the disease. Considering the more pronounced clinical presentation in children, as well as the more frequent presence of comorbidities in adults, such as hypertension and diabetes mellitus, that may independently impair kidney function regardless of the underlying disease, it can be expected that adult patients receive a diagnosis at a time when CKD has already progressed to higher stages (G3a, G3b, G4 and G5) and when eGFR values are significantly reduced [35,36]. Accordingly, in our study, the average glomerular filtration rate among adult subjects was 71 ± 37.2 mL/min, while in pediatric patients, it was 117 ± 44.3 mL/min. As a consequence of its reduction, the levels of nitrogenous waste products (creatinine and urea) in the blood increase. Additionally, serum urea levels at the time of biopsy were identified as significant predictors of both CKD progression and the progression of proteinuria in adult patients with IgA nephropathy.

In the routine evaluation of biopsy samples from patients with IgA nephropathy, the Oxford classification is applied. It takes into account acute glomerular lesions (mesangial and endocapillary hypercellularity, crescent formation), chronic glomerular changes (segmental glomerulosclerosis), and chronic tubulointerstitial lesions [9]. In our study, it was observed that changes indicative of chronicity (S1, T1, and T2) occurred more frequently in the adult population, which corresponds with the considerably more frequent diagnosis of advanced stages of CKD in this group. Additionally, we observed a moderate correlation between the Oxford classification T parameter and both decreased eGFR and advanced stages of CKD in both studied populations, as well as a correlation with higher serum urea levels in adult patients. This can also be explained by the chronic nature of these lesions, during which renal function declines and serum urea levels increase. Moreover, the literature indicates a correlation between all the glomerular parameters of the Oxford classification and proteinuria [37]. However, in our study, such a correlation was only observed with segmental glomerulosclerosis in the pediatric cohort, and with endocapillary proliferation and crescent formations in adult patients. Additionally, crescent formations in pediatric patients showed a strong correlation with decreased levels of total serum proteins and albumin, which are a direct consequence of proteinuria, as well as a moderate correlation with elevated serum urea levels within the same cohort.

According to published data, the Oxford classification parameters M1, T1, and T2 are considered independent prognostic indicators of CKD progression [20,21,22,23,24]. Due to the presence of at least moderate mesangial cell proliferation in more than 50% of glomeruli (M1) in the majority of biopsy samples, it was not possible to determine its predictive relevance. However, the results demonstrated the predictive significance of the T parameter in the progression of CKD in both study populations, as well as its predictive value in the progression of proteinuria in pediatric patients. Additionally, segmental glomerulosclerosis emerged as a predictive factor for the progression of hematuria in adult patients.

Recognizing the limitations of the Oxford classification, which primarily addresses glomerular lesions and includes only a single parameter for tubulointerstitial involvement, this study aimed to integrate Banff classification parameters, given their inclusion of vascular and more detailed tubulointerstitial changes, into the evaluation of IgA nephropathy biopsy samples, with the additional goals of assessing their correlation with clinical parameters and determining their predictive value in the progression of CKD, proteinuria, and hematuria [13]. Our study did not show significant differences in the distribution of glomerulitis (g) or mesangial matrix expansion (mm) between the study populations. However, glomerulitis correlated with elevated proteinuria levels, decreased serum albumin, as well as reduced eGFR in adult patients. Conversely, GBM double contours (cg) were observed with greater frequency in the glomerular loops of adult patients, and this parameter was identified as a significant predictive indicator for the progression of proteinuria within this cohort.

Among acute tubulointerstitial lesions, we examined tubulitis (t), total inflammation (ti), interstitial inflammation (i), and inflammation in areas of interstitial fibrosis and tubular atrophy (i-IFTA), with all parameters being more pronounced in the adult population. The literature reports a correlation between interstitial inflammation and the progression of CKD [38]. Our results demonstrated that all four parameters correlated with elevated serum urea levels, reduced eGFR, and advanced stages of CKD in adult patients. Additionally, total inflammation, interstitial inflammation, and inflammation in the area of IFTA were correlated with decreased eGFR and advanced CKD stages in the pediatric cohort, with total inflammation also showing a correlation with elevated serum urea levels in this group. Furthermore, in the pediatric population, total inflammation, interstitial inflammation, and inflammation in the area of IFTA were identified as predictors of CKD progression, while ti and i-IFTA were also predictors of proteinuria progression. In adult patients, tubulitis was identified as a predictor of both proteinuria and hematuria progression, while i-IFTA served as a predictor of proteinuria progression, although interstitial inflammation was not found to have predictive significance for CKD progression in this group.

On the other hand, Banff classification parameters that indicate chronic tubulointerstitial lesions are tubular atrophy (ct) and interstitial fibrosis (ci). The results of our study show a significantly higher frequency of these changes in adult subjects, which is consistent with the evaluated T parameter in the Oxford classification. However, the Banff classification distinguishes between the absence of these changes and the presence of mild changes (ct0 and ct1, or ci0 and ci1), all of which would correspond to a T0 score in the Oxford classification. Additionally, both parameters showed a moderate correlation with elevated serum urea levels, reduced eGFR, and advanced CKD stages in both study populations, with the exception of the correlation of tubular atrophy with eGFR and CKD stage, which did not reach statistical significance. Moreover, both parameters were identified as predictors of CKD progression in both cohorts, as well as predictors of proteinuria progression in pediatric patients. These findings are consistent with the results related to the T parameter of the Oxford classification, which reflects the same tubulointerstitial changes.

Unlike the Oxford classification, Banff classification parameters also describe vascular changes. Our study showed the more frequent and more severe fibrous intimal thickening of blood vessels (cv) in adult patients, as well as an almost exclusive occurrence of arteriolar wall hyalinization (ah, aah) in this population. Data from the literature indicate an association between fibrous intimal thickening and lower glomerular filtration rate values, as well as a connection with chronic tubulointerstitial changes [39]. Additionally, the presence of hyaline deposits in the walls of arterioles has been associated with a poorer prognosis [40]. In our study, all four parameters correlated with elevated serum urea levels, decreased eGFR, and advanced stages of CKD in adult patients. Additionally, arteriolar hyalinosis was associated with increased levels of proteinuria in the same cohort. Peritubular capillaritis and fibrous intimal thickening were further identified as significant predictors of hematuria progression in adults and proteinuria progression in pediatric patients. Naturally, such changes also occur in the context of arterial hypertension, which is one of the most common comorbidities [41].

In conclusion, pediatric patients are more commonly present with macroscopic hematuria. In contrast, adult patients exhibit reduced estimated glomerular filtration rates and a higher burden of comorbid conditions. Histopathological evaluation using both the Oxford and Banff classification systems reveals a higher prevalence of chronic lesions in the adult cohort. Notably, the Banff classification further delineates significant differences in the distribution of vascular involvement, chronically active glomerular lesions, and acute tubulointerstitial injury. All inflammatory, chronic tubulointerstitial, and vascular histopathological parameters demonstrated significant correlations with serum urea levels, reduced eGFR, and more advanced stages of CKD in adult patients. In contrast, these lesions were observed less frequently in the pediatric cohort. Notably, tubulointerstitial parameters derived from both the Oxford and Banff classifications emerged as strong and consistent predictors of CKD progression and worsening proteinuria in pediatric patients. In adult patients, however, fewer parameters exhibited predictive value. These observations collectively suggest that adult patients manifest more severe and chronic histopathological alterations, particularly within the vascular and tubulointerstitial compartments, while selected histopathological parameters, particularly those reflecting tubulointerstitial involvement, demonstrate greater predictive utility for CKD and proteinuria progression in the pediatric population. Considering that certain Banff parameters, including cg, t, ti, i, i-IFTA, ptc, and cv, which are not described by the Oxford classification, have proven to be significant predictors of outcomes in patients with IgA nephropathy, it is recommended that these parameters be incorporated into the pathohistological report when describing changes in IgA nephropathy in native kidneys.

## Figures and Tables

**Table 1:** Clinical parameters of patients with IgA nephropathy.

Clinical Parameters		Population		*p*
		Pediatric (*n* = 105)	Adult (*n* = 148)	
Sex [*n* (%)]	Male	71 (42%)	97 (58%)	0.730 *(χ*^2^ test)
	Female	34 (40%)	51 (60%)	
IgA vasculitis signs and symptoms [*n* (%)]	Absent	53 (29%)	127 (71%)	<0.001 * *(χ*^2^ test)
	Present	52 (71%)	21 (29%)	
Hematuria [*n* (%)]	Absent	14 (28%)	37 (72%)	<0.001 * *(χ*^2^ test)
	Microscopic	40 (41%)	90 (69%)	
	Macroscopic	48 (72%)	19 (28%)	
Proteinuria [*n* (%)]	Absent	15 (52%)	14 (48%)	0.383 *(χ*^2^ test)
	Subnephrotic	54 (38%)	87 (62%)	
	Nephrotic	34 (43%)	45 (57%)	
Serum total protein (g/L)		65 ± 9.8 69.5 (39–74)	65 ± 9.5 66 (33–84)	0.901 (*t*-test)
Serum albumin (g/L)		37 ± 10.1 40 (7–48)	38 ± 8.1 40 (10–51)	0.921 (MW)
Serum urea (mmol/L)		6.6 ± 3.86 5.4 (2.4–17.1)	8.8 ± 4.89 7.8 (2.0–31.5)	0.017 * (MW)
Serum creatinine (μmol/L)		91 ± 78.5 65 (29–455)	145 ± 99.6 115 (41–771)	<0.001 * (MW)
eGFR (mL/min/1.73m^2^)		117 ± 44.3 134 (17–182)	71 ± 37.2 62 (8–192)	<0.001 * (*t*-test)
Chronic kidney disease stage [*n* (%)]	G1	25 (35%)	47 (65%)	0.012 * *(χ*^2^ test)
	G2	6 (27%)	16 (73%)	
	G3a	2 (7%)	26 (93%)	
	G3b	2 (12%)	14 (88%)	
	G4	1 (7%)	14 (93%)	
	G5	0 (0%)	5 (100%)	
Hypertension [*n* (%)]	Absent	99 (60%)	65 (40%)	<0.001 * *(χ*^2^ test)
	Present	6 (7%)	83 (93%)	
Diabetes mellitus [*n* (%)]	Absent	105 (43%)	140 (57%)	0.022 * (Fisher)
	Present	0 (0%)	8 (100%)	
SGLT2 inhibitors in therapy [*n* (%)]	No	61 (58%)	45 (42%)	<0.001 * *(χ*^2^ test)
	Yes	1 (2%)	49 (98%)	
RAAS inhibitors in therapy [*n* (%)]	No	21 (72%)	8 (28%)	<0.001 * *(χ*^2^ test)
	Yes	41 (32%)	86 (68%)	
Corticosteroids in therapy [*n* (%)]	No	41 (38%)	67 (62%)	0.495 *(χ*^2^ test)
	Yes	21 (44%)	27 (56%)	

eGFR—estimated glomerular filtration rate; *—*p* < 0.050; MW—Mann–Whitney U test.

**Table 2:** Oxford classification parameters in relation to patients’ age.

Oxford Classification Parameters		Population		*p*
		Pediatric	Adult	
Mesangial hypercellularity [*n* (%)]	M0	1 (100%)	0 (0%)	0.433 (Fisher)
	M1	83 (43%)	110 (57%)	
Endocapillary hypercellularity [*n* (%)]	E0	50 (44%)	65 (56%)	0.952 *(χ*^2^ test)
	E1	34 (43%)	45 (57%)	
Segmental glomerulosclerosis/adhesion [*n* (%)]	S0	45 (53%)	40 (47%)	0.017 * *(χ*^2^ test)
	S1	39 (36%)	70 (64%)	
Tubular atrophy/interstitial fibrosis [*n* (%)]	T0	81 (56%)	63 (44%)	<0.001 * *(χ*^2^ test)
	T1	3 (8%)	37 (92%)	
	T2	0 (0%)	10 (100%)	
Cellular/fibrocellular crescents [*n* (%)]	C0	47 (39%)	73 (61%)	0.057 *(χ*^2^ test)
	C1	31 (56%)	24 (44%)	
	C2	6 (32%)	13 (68%)	

*—*p* < 0.050.

**Table 3:** Glomerular changes according to Banff classification in relation to patients’ age.

Banff Classification Parameters		Population		*p*
		Pediatric	Adult	
Glomerulitis (g) [*n* (%)]	g0	49 (39%)	77 (61%)	0.552 *(χ*^2^ test)
	g1	26 (48%)	28 (52%)	
	g2	11 (39%)	17 (61%)	
	g3	0 (0%)	1 (100%)	
GBM double contours (cg) [*n* (%)]	cg0	27 (38%)	45 (62%)	0.047 * *(χ*^2^ test)
	cg1	57 (47%)	65 (53%)	
	cg2	2 (14%)	12 (86%)	
	cg3	0 (0%)	0 (0%)	
Mesangial matrix expansion (mm) [*n* (%)]	mm0	0 (0%)	0 (0%)	0.897 *(χ*^2^ test)
	mm1	1 (50%)	1 (50%)	
	mm2	2 (33%)	4 (67%)	
	mm3	83 (41%)	118 (59%)	

GBM—glomerular basement membrane; *—*p* < 0.050.

**Table 4:** Tubulointerstitial changes according to Banff classification in relation to patients’ age.

Banff Classification Parameters		Population		*p*
		Pediatric	Adult	
Tubulitis (t) [*n* (%)]	t0	85 (47%)	96 (53%)	<0.001 * *(χ*^2^ test)
	t1	0 (0%)	18 (100%)	
	t2	1 (11%)	8 (89%)	
	t3	0 (0%)	2 (100%)	
Total inflammation (ti) [*n* (%)]	ti0	61 (63%)	36 (37%)	<0.001 * *(χ*^2^ test)
	ti1	23 (35%)	42 (65%)	
	ti2	2 (6%)	34 (94%)	
	ti3	0 (0%)	12 (100%)	
Interstitial inflammation (i) [*n* (%)]	i0	67 (54%)	57 (46%)	<0.001 * *(χ*^2^ test)
	i1	19 (25%)	58 (75%)	
	i2	0 (0%)	8 (100%)	
	i3	0 (0%)	1 (100%)	
Inflammation in the area of IFTA (i-IFTA) [*n* (%)]	i-IFTA0	78 (55%)	64 (45%)	<0.001 * *(χ*^2^ test)
	i-IFTA1	7 (14%)	45 (86%)	
	i-IFTA2	1 (7%)	14 (93%)	
	i-IFTA3	0 (0%)	1 (100%)	
Tubular atrophy (ct) [*n* (%)]	ct0	67 (72%)	26 (28%)	<0.001 * *(χ*^2^ test)
	ct1	17 (22%)	60 (78%)	
	ct2	2 (6%)	34 (94%)	
	ct3	0 (0%)	4 (100%)	
Interstitial fibrosis (ci) [*n* (%)]	ci0	70 (64%)	39 (36%)	<0.001 * *(χ*^2^ test)
	ci1	14 (30%)	32 (70%)	
	ci2	2 (5%)	38 (95%)	
	ci3	0 (0%)	15 (100%)	

IFTA—interstitial fibrosis and tubular atrophy; *—*p* < 0.050.

**Table 5:** Vascular changes according to Banff classification in relation to patients’ age.

Banff Classification Parameter		Population		*p*
		Pediatric	Adult	
Peritubular capillaritis (ptc) [*n* (%)]	ptc0	82 (45%)	100 (55%)	0.022 * *(χ*^2^ test)
	ptc1	2 (13%)	13 (87%)	
	ptc2	2 (17%)	10 (83%)	
	ptc3	0 (0%)	1 (100%)	
Vascular fibrous intimal thickening (cv) [*n* (%)]	cv0	83 (46%)	99 (54%)	0.004 * *(χ*^2^ test)
	cv1	2 (25%)	6 (75%)	
	cv2	0 (0%)	9 (100%)	
	cv3	1 (9%)	10 (91%)	
Arteriolar hyalinosis (ah) [*n* (%)]	ah0	84 (51%)	82 (49%)	<0.001 * *(χ*^2^ test)
	ah1	1 (6%)	17 (94%)	
	ah2	1 (8%)	11 (92%)	
	ah3	0 (0%)	14 (100%)	
Hyaline arteriolar thickening (aah) [*n* (%)]	aah0	84 (51%)	82 (49%)	<0.001 * *(χ*^2^ test)
	aah1	1 (33%)	2 (67%)	
	aah2	0 (0%)	27 (100%)	
	aah3	1 (7%)	13 (93%)	

*—*p* < 0.050.

**Table 6:** Correlations of clinical data collected at the time of biopsy with Oxford and Banff pathohistological parameters in pediatric patients.

	Hematuria	Proteinuria	s Total Protein	s Albumin	s Urea	eGFR	CKD Stage
Mesangial hypercellularity (M)	r_s_ = 0.178 *p* = 0.110	r_s_ = 0.178 *p* = 0.108	N/A	N/A	N/A	N/A	N/A
Endocapillary hypercellularity (E)	r_s_ = 0.049 *p* = 0.664	r_s_ = 0.079 *p* = 0.478	r_s_ = −0.525 *p* = 0.119	r_s_ = −0.437 *p* = 0.156	r_s_ = 0.157 *p* = 0.546	r_s_ = −0.068 *p* = 0.737	r_s_ = 0.249 *p* = 0.210
Segmental glomerulosclerosis (S)	r_s_ = 0.063 *p* = 0.575	r_s_ = 0.280 *p* = 0.010 *	r_s_ = −0.263 *p* = 0.463	r_s_ = −0.077 *p* = 0.812	r_s_ = 0.343 *p* = 0.178	r_s_ = −0.148 *p* = 0.463	r_s_ = 0.125 *p* = 0.533
Tubular atrophy/Interstitial fibrosis (T)	r_s_ = 0.081 *p* = 0.468	r_s_ = 0.042 *p* = 0.709	N/A	N/A	r_s_ = 0.408 *p* = 0.104	r_s_ = −0.418 *p* = 0.030 *	r_s_ = 0.500 *p* = 0.008 *
Cellular and fibrocellular crescents (C)	r_s_ = −0.074 *p* = 0.510	r_s_ = 0.202 *p* = 0.067	r_s_ = −0.775 *p* = 0.008 *	r_s_ = −0.701 *p* = 0.011 *	r_s_ = 0.548 *p* = 0.023 *	r_s_ = −0.221 *p* = 0.267	r_s_ = 0.116 *p* = 0.565
Glomerulitis (g)	r_s_ = 0.042 *p* = 0.707	r_s_ = 0.100 *p* = 0.364	r_s_ = −0.531 *p* = 0.114	r_s_ = −0.109 *p* = 0.722	r_s_ = 0.156 *p* = 0.563	r_s_ = 0.074 *p* = 0.713	r_s_ = 0.169 *p* = 0.398
GBM double contours (cg)	r_s_ = −0.042 *p* = 0.706	r_s_ = 0.105 *p* = 0.339	r_s_ = −0.263 *p* = 0.463	r_s_ = −0.220 *p* = 0.470	r_s_ = −0.084 *p* = 0.757	r_s_ = 0.251 *p* = 0.207	r_s_ = −0.220 *p* = 0.270
Mesangial matrix expansion (mm)	r_s_ = 0.178 *p* = 0.104	r_s_ = 0.046 *p* = 0.675	r_s_ = −0.175 *p* = 0.628	r_s_ = −0.387 *p* = 0.192	r_s_ = 0.196 *p* = 0.467	r_s_ = −0.189 *p* = 0.345	r_s_ = 0.136 *p* = 0.500
Tubulitis (t)	r_s_ = −0.067 *p* = 0.546	r_s_ = −0.037 *p* = 0.738	N/A	N/A	N/A	r_s_ = −0.277 *p* = 0.162	r_s_ = 0.347 *p* = 0.077
Total inflammation (ti)	r_s_ = 0.104 *p* = 0.345	r_s_ = 0.041 *p* = 0.708	N/A	r_s_ = −0.464 *p* = 0.110	r_s_ = 0.575 *p* = 0.020 *	r_s_ = −0.691 *p* < 0.001 *	r_s_ = 0.738 *p* < 0.001 *
Interstitial inflammation (i)	r_s_ = 0.140 *p* = 0.204	r_s_ = −0.089 *p* = 0.416	N/A	N/A	r_s_ = 0.420 *p* = 0.105	r_s_ = −0.551 *p* = 0.003 *	r_s_ = 0.564 *p* = 0.002 *
Inflammation in the area of IFTA (i-IFTA)	r_s_ = 0.125 *p* = 0.259	r_s_ = 0.040 *p* = 0.718	N/A	N/A	r_s_ = 0.420 *p* = 0.105	r_s_ = −0.418 *p* = 0.030 *	r_s_ = 0.500 *p* = 0.008 *
Tubular atrophy (ct)	r_s_ = 0.196 *p* = 0.074	r_s_ = −0.053 *p* = 0.628	r_s_ < 0.001 *p* = 1.000	r_s_ = −0.147 *p* = 0.632	r_s_ = 0.678 *p* = 0.004 *	r_s_ = −0.333 *p* = 0.090	r_s_ = 0.357 *p* = 0.068
Interstitial fibrosis (ci)	r_s_ = −0.074 *p* = 0.505	r_s_ = 0.019 *p* = 0.862	N/A	r_s_ = −0.464 *p* = 0.110	r_s_ = 0.575 *p* = 0.020 *	r_s_ = −0.390 *p* = 0.044 *	r_s_ = 0.541 *p* = 0.004 *
Peritubular capillaritis (ptc)	r_s_ = 0.126 *p* = 0.252	r_s_ = 0.101 *p* = 0.359	N/A	N/A	r_s_ = 0.420 *p* = 0.105	r_s_ = −0.417 *p* = 0.031 *	r_s_ = 0.500 *p* = 0.008 *
Vascular fibrous intimal thickening (cv)	r_s_ = 0.019 *p* = 0.862	r_s_ = −0.148 *p* = 0.176	N/A	r_s_ = 0.193 *p* = 0.527	N/A	r_s_ = −0.025 *p* = 0.901	r_s_ = −0.136 *p* = 0.500
Arteriolar hyalinosis (ah)	r_s_ = −0.246 *p* = 0.024 *	r_s_ = −0.153 *p* = 0.162	N/A	N/A	r_s_ = −0.420 *p* = 0.105	r_s_ = 0.327 *p* = 0.096	r_s_ = −0.136 *p* = 0.500
Hyaline arteriolar thickening (aah)	r_s_ = −0.246 *p* = 0.024 *	r_s_ = −0.153 *p* = 0.162	N/A	N/A	r_s_ = −0.420 *p* = 0.105	r_s_ = 0.327 *p* = 0.096	r_s_ = −0.136 *p* = 0.500

s—serum; eGFR—estimated glomerular filtration rate; CKD—chronic kidney disease; r_s_—Spearman’s rank correlation coefficient; N/A—not applicable; *—*p* < 0.050.

**Table 7:** Correlations of clinical data collected at the time of biopsy with Oxford and Banff pathohistological parameters in adult patients.

	Hematuria	Proteinuria	s Total Protein	s Albumin	s Urea	eGFR	CKD Stage
Mesangial hypercellularity (M)	N/A	N/A	N/A	N/A	N/A	N/A	N/A
Endocapillary hypercellularity (E)	r_s_ = 0.142 *p* = 0.140	r_s_ = 0.191 *p* = 0.045 *	r_s_ = −0.210 *p* = 0.065	r_s_ = −0.188 *p* = 0.095	r_s_ = 0.101 *p* = 0.347	r_s_ = −0.271 *p* = 0.006 *	r_s_ = 0.245 *p* = 0.013 *
Segmental glomerulosclerosis (S)	r_s_ = −0.011 *p* = 0.906	r_s_ = 0.056 *p* = 0.562	r_s_ = −0.005 *p* = 0.968	r_s_ = 0.025 *p* = 0.826	r_s_ = 0.055 *p* = 0.612	r_s_ = −0.234 *p* = 0.019 *	r_s_ = 0.174 *p* = 0.080
Tubular atrophy/interstitial fibrosis (T)	r_s_ = −0.032 *p* = 0.740	r_s_ = 0.163 *p* = 0.088	r_s_ = −0.134 *p* = 0.241	r_s_ = −0.222 *p* = 0.047 *	r_s_ = 0.559 *p* < 0.001 *	r_s_ = −0.636 *p* < 0.001 *	r_s_ = 0.667 *p* < 0.001 *
Cellular and fibrocellular crescents (C)	r_s_ = 0.182 *p* = 0.058	r_s_ = 0.194 *p* = 0.042 *	r_s_ = −0.166 *p* = 0.146	r_s_ = −0.222 *p* = 0.048 *	r_s_ = 0.125 *p* = 0.247	r_s_ = −0.327 *p* = 0.001 *	r_s_ = 0.309 *p* = 0.002 *
Glomerulitis (g)	r_s_ = 0.120 *p* = 0.185	r_s_ = 0.192 *p* = 0.033 *	r_s_ = −0.181 *p* = 0.091	r_s_ = −0.286 *p* = 0.007 *	r_s_ = 0.161 *p* = 0.113	r_s_ = −0.318 *p* = 0.001 *	r_s_ = 0.311 *p* = 0.001 *
GBM double contours (cg)	r_s_ = 0.006 *p* = 0.949	r_s_ = −0.131 *p* = 0.151	r_s_ = −0.021 *p* = 0.845	r_s_ = 0.035 *p* = 0.751	r_s_ = −0.162 *p* = 0.110	r_s_ = 0.065 *p* = 0.496	r_s_ = −0.035 *p* = 0.716
Mesangial matrix expansion (mm)	r_s_ = 0.033 *p* = 0.718	r_s_ = 0.160 *p* = 0.078	r_s_ = −0.065 *p* = 0.547	r_s_ = 0.119 *p* = 0.270	r_s_ = 0.068 *p* = 0.501	r_s_ = −0.106 *p* = 0.262	r_s_ = 0.111 *p* = 0.238
Tubulitis (t)	r_s_ = −0.025 *p* = 0.781	r_s_ = 0.042 *p* = 0.645	r_s_ = −0.039 *p* = 0.719	r_s_ = −0.105 *p* = 0.332	r_s_ = 0.331 *p* = 0.001 *	r_s_ = −0.418 *p* < 0.001 *	r_s_ = 0.456 *p* < 0.001 *
Total inflammation (ti)	r_s_ = −0.056 *p* = 0.540	r_s_ = 0.066 *p* = 0.464	r_s_ = 0.021 *p* = 0.847	r_s_ = −0.104 *p* = 0.335	r_s_ = 0.554 *p* < 0.001 *	r_s_ = −0.639 *p* < 0.001 *	r_s_ = 0.659 *p* < 0.001 *
Interstitial inflammation (i)	r_s_ = 0.042 *p* = 0.646	r_s_ = 0.127 *p* = 0.159	r_s_ = −0.043 *p* = 0.691	r_s_ = −0.113 *p* = 0.296	r_s_ = 0.405 *p* < 0.001 *	r_s_ = −0.538 *p* < 0.001 *	r_s_ = 0.547 *p* < 0.001 *
Inflammation in the area of IFTA (i-IFTA)	r_s_ = −0.044 *p* = 0.626	r_s_ = 0.051 *p* = 0.570	r_s_ = −0.013 *p* = 0.904	r_s_ = −0.132 *p* = 0.221	r_s_ = 0.578 *p* < 0.001 *	r_s_ = −0.667 *p* < 0.001 *	r_s_ = 0.655 *p* < 0.001 *
Tubular atrophy (ct)	r_s_ = 0.038 *p* = 0.678	r_s_ = 0.057 *p* = 0.530	r_s_ = −0.038 *p* = 0.724	r_s_ = −0.122 *p* = 0.258	r_s_ = 0.431 *p* < 0.001 *	r_s_ = −0.600 *p* < 0.001 *	r_s_ = 0.610 *p* < 0.001 *
Interstitial fibrosis (ci)	r_s_ = −0.021 *p* = 0.820	r_s_ = 0.069 *p* = 0.444	r_s_ = −0.055 *p* = 0.610	r_s_ = −0.141 *p* = 0.191	r_s_ = 0.569 *p* < 0.001 *	r_s_ = −0.640 *p* < 0.001 *	r_s_ = 0.659 *p* < 0.001 *
Peritubular capillaritis (ptc)	r_s_ = −0.092 *p* = 0.309	r_s_ = 0.055 *p* = 0.541	r_s_ = −0.050 *p* = 0.642	r_s_ = −0.141 *p* = 0.191	r_s_ = 0.283 *p* = 0.005 *	r_s_ = −0.401 *p* < 0.001 *	r_s_ = 0.384 *p* < 0.001 *
Vascular fibrous intimal thickening (cv)	r_s_ = 0.040 *p* = 0.657	r_s_ = 0.115 *p* = 0.202	r_s_ = −0.102 *p* = 0.344	r_s_ = −0.177 *p* = 0.100	r_s_ = 0.200 *p* = 0.047 *	r_s_ = −0.355 *p* < 0.001 *	r_s_ = 0.362 *p* < 0.001 *
Arteriolar hyalinosis (ah)	r_s_ = −0.143 *p* = 0.112	r_s_ = 0.200 *p* = 0.026 *	r_s_ = −0.094 *p* = 0.386	r_s_ = −0.188 *p* = 0.079	r_s_ = 0.351 *p* < 0.001 *	r_s_ = −0.294 *p* = 0.002 *	r_s_ = 0.259 *p* = 0.005 *
Hyaline arteriolar thickening (aah)	r_s_ = −0.164 *p* = 0.069	r_s_ = 0.189 *p* = 0.036 *	r_s_ = −0.077 *p* = 0.475	r_s_ = −0.170 *p* = 0.113	r_s_ = 0.331 *p* = 0.001 *	r_s_ = −0.285 *p* = 0.002 *	r_s_ = 0.250 *p* = 0.007 *

s—serum; eGFR—estimated glomerular filtration rate; CKD—chronic kidney disease; N/A—not applicable; r_s_—Spearman’s rank correlation coefficient; *—*p* < 0.050.

**Table 8:** The incidence of the outcomes in pediatric and adult IgA nephropathy.

		Population		*p*
		Pediatric	Adult	
CKD progression [*n* (%)]	progression	2 (6%)	32 (94%)	0.036 * *(χ*^2^ test)
	without change	13 (29%)	32 (71%)	
	regression	4 (19%)	17 (81%)	
Proteinuria progression [*n* (%)]	progression	5 (23%)	17 (77%)	0.196 *(χ*^2^ test)
	without change	11 (48%)	12 (52%)	
	regression	35 (40%)	52 (60%)	
Hematuria progression [*n* (%)]	progression	1 (14%)	6 (86%)	0.155 *(χ*^2^ test)
	without change	18 (32%)	38 (68%)	
	regression	34 (44%)	43 (56%)	

CKD—chronic kidney disease; *—*p* < 0.050.

**Table 9:** Clinical predictors of the outcome in pediatric and adult onset IgA nephropathy in native kidneys.

	Pediatric Population			Adult Population		
	CKD Progression	Proteinuria Progression	Hematuria Progression	CKD Progression	Proteinuria Progression	Hematuria Progression
Sex	*p* = 0.599	*p* = 0.144	*p* = 0.237	*p* = 0.313	*p* = 0.430	*p* = 0.435
IgA vasculitis signs and symptoms	*p* = 0.397	*p* = 0.253	*p* = 0.480	*p* = 0.792	*p* = 0.195	*p* = 0.464
Hematuria	*p* = 0.558	*p* = 0.368	N/A	*p* = 0.116	*p* = 0.040 *	N/A
Proteinuria	*p* = 0.500	N/A	*p* = 0.564	*p* = 0.244	N/A	*p* = 0.418
Serum total protein	N/A	N/A	N/A	*p* = 0.735	*p* = 0.293	*p* = 0.317
Serum albumin	N/A	*p* = 0.527	N/A	*p* = 0.273	*p* = 0.603	*p* = 0.617
Serum urea	*p* = 0.127	*p* = 0.090	N/A	*p* = 0.008 *	*p* < 0.001 *	*p* = 0.429
eGFR	*p* = 0.219	*p* = 0.965	N/A	*p* = 0.041 *	*p* = 0.019 *	*p* = 0.237
Hypertension	*p* = 0.808	*p* = 0.710	N/A	*p* = 0.348	*p* = 0.590	*p* = 0.546
Diabetes mellitus	N/A	N/A	N/A	*p* = 0.836	*p* = 0.776	*p* = 0.272
SGLT2 inhibitors	N/A	N/A	N/A	*p* = 0.332	*p* = 0.730	*p* = 0.538
RAAS inhibitors	*p* = 0.431	*p* = 0.878	*p* = 0.480	*p* = 0.038 *	*p* = 0.976	*p* = 0.582
Corticosteroids	*p* = 0.327	*p* = 0.631	*p* = 0.655	*p* = 0.033 *	*p* = 0.439	*p* = 0.077

CKD—chronic kidney disease; N/A—not applicable; *—*p* < 0.050; eGFR—estimated glomerular filtration rate.

**Table 10:** Pathohistological predictors of the outcome in pediatric and adult onset IgA nephropathy in native kidneys.

	Pediatric Population			Adult Population		
	CKD Progression	proteinuria Progression	Hematuria Progression	CKD Progression	Proteinuria Progression	Hematuria Progression
**Oxford classification parameters**						
Mesangial hypercellularity (M)	N/A	N/A	N/A	N/A	N/A	N/A
Endocapillary hypercellularity (E)	*p* = 0.323	*p* = 0.313	*p* = 0.513	*p* = 0.194	*p* = 0.260	*p* = 0.417
Segmental glomerulosclerosis (S)	*p* = 0.093	*p* = 0.275	*p* = 0.414	*p* = 0.145	*p* = 0.328	*p* = 0.025 *
Tubular atrophy/Interstitial fibrosis (T)	*p* < 0.001 *	*p* = 0.001 *	N/A	*p* = 0.042 *	*p* = 0.207	*p* = 0.305
Cellular and fibrocellular crescents (C)	*p* = 0.886	*p* = 0.710	*p* = 0.472	*p* = 0.984	*p* = 0.867	*p* = 0.837
**Banff classification parameters**						
Glomerulitis (g)	*p* = 0.506	*p* = 0.385	*p* = 0.700	*p* = 0.378	*p* = 0.386	*p* = 0.882
GBM double contours (cg)	*p* = 0.531	*p* = 0.969	N/A	*p* = 0.777	*p* = 0.006 *	*p* = 0.834
Mesangial matrix expansion (mm)	N/A	*p* = 0.879	*p* = 0.763	*p* = 0.713	*p* = 0.783	*p* = 0.589
Tubulitis (t)	N/A	N/A	N/A	*p* = 0.893	*p* = 0.038 *	*p* = 0.006 *
Total inflammation (ti)	*p* = 0.001 *	*p* < 0.001 *	*p* = 0.882	*p* = 0.052	*p* = 0.070	*p* = 0.867
Interstitial inflammation (i)	*p* < 0.001 *	*p* = 0.090	*p* = 0.655	*p* = 0.127	*p* = 0.448	*p* = 0.876
Inflammation in the area of IFTA (i-IFTA)	*p* < 0.001 *	*p* < 0.001 *	N/A	*p* = 0.067	*p* < 0.001 *	*p* = 0.979
Tubular atrophy (ct)	*p* = 0.001 *	*p* < 0.001 *	N/A	*p* = 0.047 *	*p* = 0.232	*p* = 0.753
Interstitial fibrosis (ci)	*p* = 0.001 *	*p* < 0.001 *	N/A	*p* = 0.002 *	*p* = 0.099	*p* = 0.442
Peritubular capillaritis (ptc)	*p* < 0.001 *	*p* < 0.001 *	N/A	*p* = 0.356	*p* = 0.776	*p* = 0.005 *
Vascular fibrous intimal thickening (cv)	*p* = 0.789	*p* = 0.025 *	N/A	*p* = 0.305	*p* = 0.787	*p* = 0.007 *
Arteriolar hyalinosis (ah)	*p* = 0.789	*p* = 0.716	N/A	*p* = 0.523	*p* = 0.488	*p* = 0.615
Hyaline arteriolar thickening (aah)	*p* = 0.789	*p* = 0.716	N/A	*p* = 0.021 *	*p* = 0.284	*p* = 0.934

CKD—chronic kidney disease; N/A—not applicable; *—*p* < 0.050; IFTA—interstitial fibrosis and tubular atrophy.

## Data Availability

The original contributions presented in this report are included in the article. Further enquiries can be directed to the corresponding authors.
